# MR imaging of the quadriceps femoris tendon: distal tear characterization and clinical significance of rupture types

**DOI:** 10.1007/s00330-021-07912-y

**Published:** 2021-04-16

**Authors:** Anna L. Falkowski, Jon A. Jacobson, Michael T. Hirschmann, Vivek Kalia

**Affiliations:** 1 Department of Radiology, University of Michigan, 1500 East Medical Center Drive, Ann Arbor, MI 48103 USA; 2 Department of Radiology, Balgrist University Hospital, University of Zurich, Forchstrasse 340, 8008 Zurich, Switzerland; 3 Department of Orthopaedic Surgery and Traumatology, Kantonsspital Baselland (Bruderholz, Liestal, Laufen), 4101 Bruderholz, Switzerland

**Keywords:** Tendons, Rupture, Knee, Magnetic resonance imaging, Quadriceps muscle

## Abstract

**Objective:**

To characterize quadriceps femoris tendon tears on magnetic resonance (MR) imaging regarding tear extent, location, and presence of bony avulsion.

**Materials and methods:**

IRB approval was obtained and informed consent was waived for this retrospective case series. Electronic medical records from all patients in our hospital system were searched for keywords: knee MR imaging, and quadriceps tendon rupture or tear. MRI studies were randomized and independently evaluated by two fellowship-trained musculoskeletal radiologists. MR imaging was used to characterize each individual quadriceps tendon as having tendinosis, tear (location, partial versus complete, size, and retraction distance), and bony avulsion. Knee radiographs were reviewed for presence or absence of bony avulsion. Descriptive statistics and inter-reader reliability (Cohen’s Kappa and Wilcoxon-signed-rank test) were calculated.

**Results:**

Fifty-two patients with 53 quadriceps tears were evaluated (45 males, 7 females; mean age: 51 ± 13 years). The vastus intermedius (VI) tendon more often incurred a partial rather than a complete tear (39.6% vs. 37.7%), while the rectus femoris (RF), vastus medialis (VM), and vastus lateralis (VL) incurred complete tears more commonly (64.2–66%). Subjects with bony avulsion on radiographs had higher-grade tears of the RF, VM, and VL tears (*p* = 0.020–0.043) but not the VI. Most tendons tore at or immediately proximal to the patella (84.8–93.6%). Gaps in retracted torn tendons measured between 2.3 and 2.7 cm. Inter-reader reliability was substantial to almost perfect (κ = 0.624–0.953).

**Conclusion:**

Quadriceps femoris tendon tears most commonly involve the RF or VL/VM layers usually in proximity to the patella. A bony avulsion correlates with a more extensive tear.

**Key Points:**

*• Quadriceps femoris tendon tears most commonly involve the rectus femoris or vastus lateralis/vastus medialis layers.*

*• A rupture of the quadriceps femoris tendon usually occurs in proximity to the patella.*

*• A bony avulsion of the patella correlates with a more extensive tear of the superficial and middle layers of the quadriceps tendon.*

## Introduction

Rupture of the quadriceps tendon is the second most common injury to the extensor mechanism following patellar fracture and is usually due to indirect low-energy trauma [1–3]. Anatomical areas of hypovascularity within the tendon may be predisposed to rupture [4]. Accurate clinical diagnosis tailors treatment decisions and is crucial to avoid quadriceps tendon contraction resulting in fibrosis and loss of elasticity of the muscle fibers [1, 5–8].

The distal quadriceps femoris tendon consists of three layers: the RF forms the superficial layer, the middle layer consists of the combined VM and VL tendons, and the deep layer consists of the VI tendon [9]. The muscle fibers from the quadriceps blend with its tendinous portion approximately 3 cm proximal to the patella to form the layered tendon [10]. The most superficial layer of the RF extends distally over the patella to the patellar tendon, termed the prepatellar quadriceps continuation [11]. Imaging, such as radiography, ultrasound, and magnetic resonance (MR) imaging, may be used to evaluate clinically suspected quadriceps tears [3, 8, 12]. Imaging might help to characterize quadriceps tendon tears as complete versus incomplete rupture, to assess each layer of the quadriceps tendon individually, as well as to distinguish an avulsion from an intratendinous tear [5, 9]. Familiarity with MR imaging appearances of quadriceps tears is essential, as accurate characterization of tear pattern along with a patient’s clinical context allows surgeons to tailor therapy optimally.

While the literature does describe the common sites of quadriceps tears, there are significant discrepancies with regard to reported location of tears (directly at the bone insertion on the patella versus intratendinous) [1, 4, 6, 13–16] and tear extent (superficial versus the deeper layers of the quadriceps tendon) [1, 4, 14, 17, 18]. In addition, studies describing rupture sites have a small number of cases and tend to be cited in review articles, perpetuating these hypotheses as common findings. An example is the study by Zeiss et al which examined the anatomical layers of the quadriceps tendon [9]. This study additionally evaluated only five abnormal quadriceps tendons, without concluding which tendon components tend to rupture most commonly. Thus, the purpose of our study is to evaluate and characterize quadriceps femoris tendon tears and add to the body of knowledge in this domain. Such information could lead to more precise diagnosis and description of findings for the referring physician’s benefit.

## Materials and methods

### Inclusion and exclusion criteria

Institutional Review Board approval was obtained for this Health Insurance Portability and Accountability Act–compliant study, and informed consent was waived to retrospectively evaluate MR images of the knee with quadriceps tendon ruptures. The Electronic Medical Record Search Engine (EMERSE) [19] was used to search in a database of over 2.3 million patients. This database consists of all electronic medical records of patients’, including patients’ history, referrals, laboratory values, and radiologic reports. The database was searched for key words “quadriceps tendon rupture,” “quadriceps tendon tear,” “knee MRI,” and any combination of these terms. Exclusion criteria included denial of these terms (e.g., “no quadriceps tendon rupture”), postoperative cases of knee surgeries for treatment of quadriceps tendon pathologies or total knee arthroplasties (due to possible metal artifacts obstructing the diagnosis), and cases with no available imaging. Inclusion criteria were an MR imaging exam of the knee with a partial or complete rupture of the quadriceps tendon. Presence of radiographic studies of the knee of interest was not a mandatory criterion for inclusion. However, if a radiograph was performed this was also evaluated in the study.

### Imaging

MR images were acquired in a single hospital system on five scanners from two different vendors (Philips Medical Systems; General Electric) at either 1.5 T (Philips Medical System; General Electric) or 3.0 T (Philips Medical Systems). The standard MR protocol consisted of sagittal proton-density (PD) fat-saturated and sagittal PD non-fat-saturated images, coronal PD fat-saturated images, axial T2 fat-saturated images, and axial T1 non-fat-saturated images. In addition, if available, lateral knee radiographs of the corresponding knee were evaluated for a possible bony avulsion.

### Image review

Images were randomized and evaluated independently by two fellowship-trained musculoskeletal radiologists with 6 and 7 years of experience on Food and Drug Administration–approved PACS workstations (McKesson Technology Solutions). Both readers were blinded to the patients’ treatment and history, as well as to each other’s findings. Midsagittal and paramidline sagittal MR images were used for measurements; however, axial and coronal MR images were used as needed to clarify imaging findings, e.g., tendon involvement. On MR imaging, the following tear characteristics were evaluated for each individual quadriceps femoris tendon component (rectus femoris, vastus medialis, vastus lateralis, and vastus intermedius): tendon quality (normal, tendinosis, partial tear, or complete tear) and location of the tear, if present (bone avulsion, at bone insertion, proximal to the bone insertion or at the myotendinous junction). Tendinosis was defined as an increase in signal intensity of the tendon and tendon thickening [20]. Partial tears were defined as a partial disruption of the tendon, while the rest of the tendon was still intact; for this evaluation, all three imaging planes were used. For the evaluation of the conjoined vastus medialis and lateralis tendon, each tendon was assessed separately. Characterization of a tear was accompanied by measurement of the length of the remaining tendon stump and the tendon retraction for each tendon. Also, the quadriceps tendon continuation was evaluated qualitatively (normal or abnormal).

If radiographs were available, the presence or absence of a bony avulsion fragment on knee radiographs was documented. Lateral radiographs were used for measurements, as well as the distance of the fragment to the patella. In addition, lateral radiographs were used to evaluate the number of fragments (solitary or multiple), and the size of the largest avulsed fragment. Presence or absence of enthesophytes was documented, as well as avulsion of these (with and/or without bony involvement).

### Patient records

Patients’ medical records were reviewed, regarding conservative versus surgical treatment. Surgical techniques were documented and surgical findings were reviewed, regarding partial versus complete tears and their correlation to MR imaging.

In addition, patients’ medical records were reviewed for presence or absence of additional disorders (diabetes type 1 or 2, gout, collagen disorders, and vascular disorders). The trauma mechanism (low-energy versus high-energy trauma) was documented.

### Statistical analysis

Statistical analyses were performed with Microsoft Excel’s data package and SPSS software (version 22.0). Descriptive statistics were calculated (mean, standard deviation, range, and percentage). Patients with bone avulsions (*n* = 24) were compared to those without bony avulsions (*n* = 29) using Student *t* tests. Inter-reader reliability was calculated using Cohen’s Kappa and Wilcoxon-signed-rank test. Strength of inter-observer agreement (κ) was characterized as follows: poor (κ < 0.1), slight (0.1 ≤ κ ≤ 0.2), fair (0.2 < κ ≤ 0.4), moderate (0.4 < κ ≤ 0.6), substantial (0.6 < κ ≤ 0.8), and almost perfect (0.8 < κ ≤ 1). *p* < 0.05 was considered indicative of a statistically significant difference.

## Results

### Database search

**Fig. 1:**
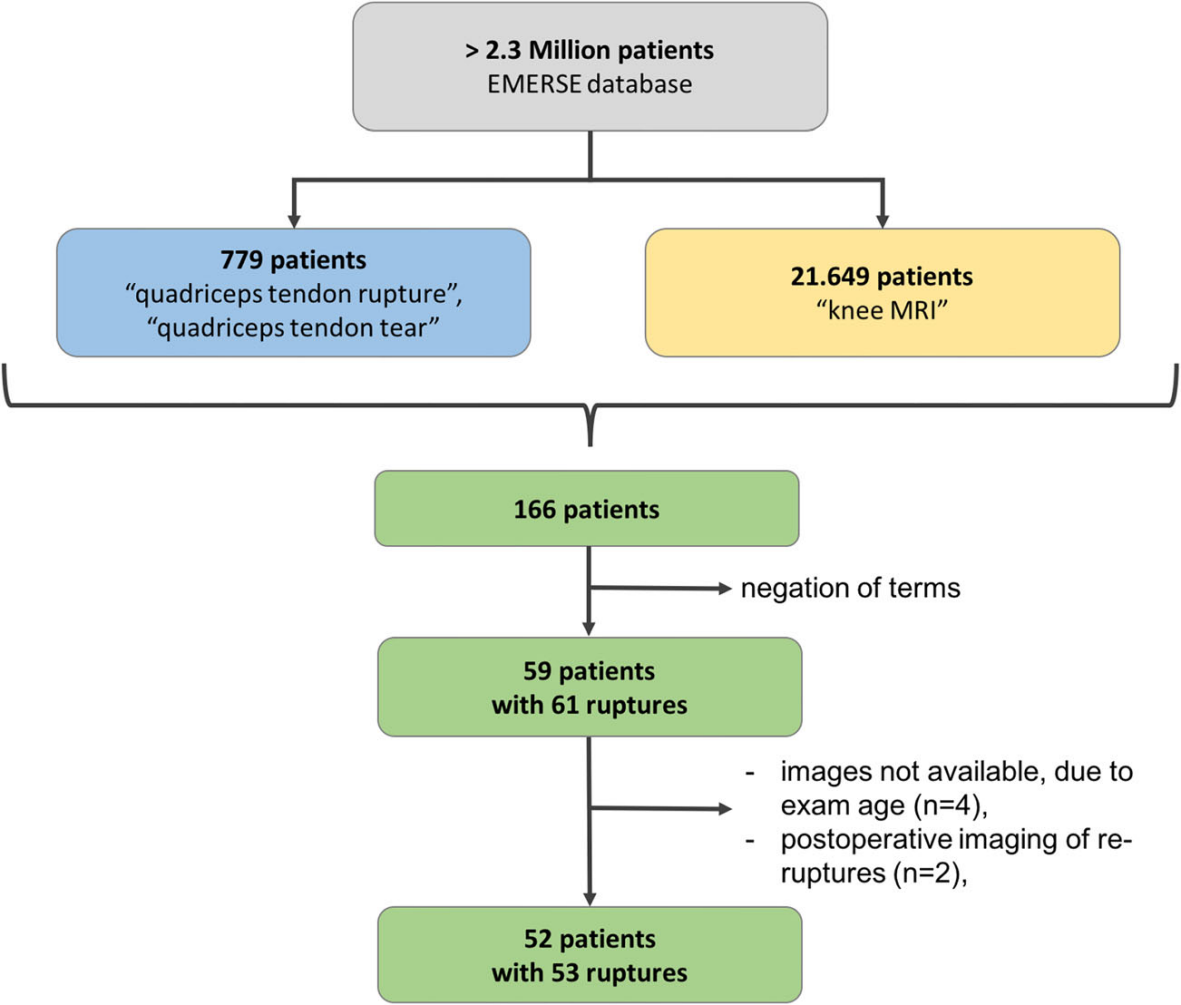
Chart shows the keyword search performed to identify patients with quadriceps tendon rupture or tear who underwent knee MRI. Blue color denotes patients with the term “quadriceps tendon rupture” or “quadriceps tendon tear” in their medical record, yellow color denotes patients who underwent knee MRI, and green color are the net patients (*n* = 166) who underwent knee MRI with the above-mentioned quadriceps tendon terms in their MRI report. Patients with denial of these terms (e.g., “no quadriceps tendon rupture”), and additional listed criteria were excluded. Thus, 52 patients with 53 quadriceps tendon ruptures remained for evaluation

We searched the EMERSE database of over 2.3 million patients for the key terms “quadriceps tendon rupture” and/or “quadriceps tendon tear,” which yielded 779 patients. In addition, the key term “knee MRI” yielded 21,649 patients. Merging these groups revealed an overlap of 166 patients. All knee MRI reports (*n* = 171) of the 166 patients were searched to exclude patients without quadriceps tendon rupture or tear. A total of 58 patients (comprising a total of 60 quadriceps tendon ruptures) had quadriceps tendon ruptures described in their MR imaging reports. Five cases in four patients were excluded due to images being unavailable to interpret. In addition, 2 cases of re-rupture after postoperative fixation of the quadriceps tendon were excluded. Thus, the final cohort we evaluated consisted of 52 patients with 53 MR imaging exams of quadriceps tendon ruptures (Fig. 1). MR examinations of the included patients’ involved knees were performed between August 2000 and May 2018. All included cases but one had knee radiography available for evaluation.

### Demographics

Of the 52 evaluated patients, 45 were males (87.5%) and 7 were females (12.5%). The average age of the patients was 51 ± 13 (range 16 to 83) years. The cases consisted of 28 right and 25 left knees. One patient experienced quadriceps tendon ruptures bilaterally, however three years apart. On the 53 MR imaging exams, we found in total 183 tendon ruptures (including partial and complete) of all subtendons (RF, VM, VL, and VI) of the 53 quadriceps femoris tendons.

### Tendon quality evaluation

The rectus femoris (RF), vastus medialis (VM), vastus lateralis (VL), and vastus intermedius (VI) tendons were normal in 3.8%, 7.5%, 3.8%, and 18.9% respectively. Observers reported tendinosis in 7.5%, 5.7%, 3.8%, and 3.8% respectively. The detailed distribution of tendon quality is listed in Table 1. The VI tendon more often incurred a partial rather than a complete tear (21/53 or 39.6% vs. 20/53 or 37.7%), while the RF, VM, and VL incurred complete tears more commonly (64.2–66.0%) (Table 1). Inter-reader reliability was substantial to almost perfect for the tendon quality evaluation (κ = 0.736–0.858).

**Table 1:** Tendon quality for each quadriceps tendon is shown as a percentage of the total in categories of normal, tendinosis, partial tear, or complete tear. Cohen’s Kappa inter-reader agreement is listed in the last row

	Rectus femoris	Vastus medialis	Vastus lateralis	Vastus intermedius
Normal	3.8% (2/53)	7.5% (4/53)	3.8% (2/53)	18.9% (10/53)
Tendinosis	7.5% (4/53)	5.7% (3/53)	3.8% (2/53)	3.8% (2/53)
Partial tear	22.6% (12/53)	20.8% (11/53)	28.3% (15/53)	39.6% (21/53)
Complete tear	66.0% (35/53)	66.0% (35/53)	64.2% (34/53)	37.7% (20/53)
Inter-reader agreement	κ = 0.858	κ = 0.812	κ = 0.827	κ = 0.736

**Fig. 2:**
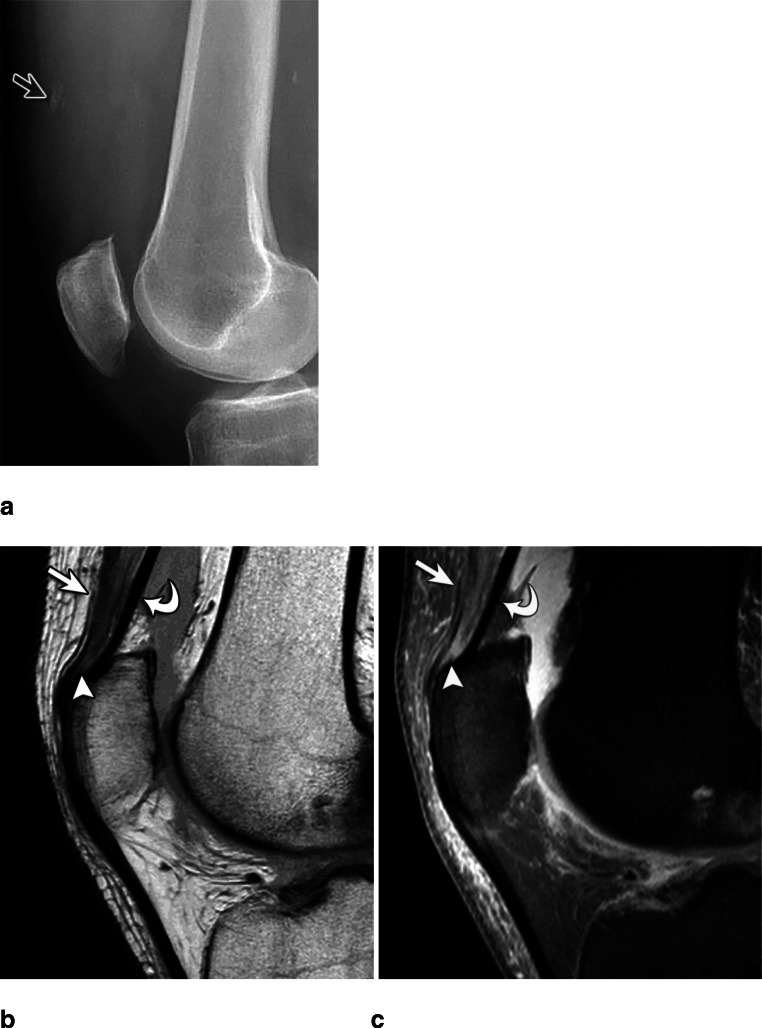
Sixty-year-old male with quadriceps tendon tear and bone avulsion. Radiograph (**a**) shows a bone avulsion (open arrow). Sagittal intermediate-weighted MR image with (**b**) and without (**c**) fat saturation shows complete tears of the rectus femoris (arrow), as well as the combined vastus medialis and lateralis (arrowhead) tendon. The vastus intermedius is normal (curved arrow). The patient was treated conservatively

A bony avulsion was present in 49% (26/53) of cases and showed mostly multiple (69%; 18/26) rather than single fragments (31%; 8/26) (Fig. 2a). The size of the largest avulsed fragment ranged from 0.3 to 2.3 cm (average 1.0 cm). Patients who showed bony avulsion on radiographs had higher-grade tears (i.e., complete or partial tears) of the RF, VM, and VL (*p* = 0.020–0.043), but not of the VI (Fig. 2 and Table 2). Inter-reader reliability was almost perfect for bone avulsions (κ = 0.953) and number of avulsed fragments (κ = 0.909). Enthesophytes were present in 66% (35/53) of cases, and were avulsed in 48% (17/35). In 17% (6/35) of enthesophytes, a bony avulsion of the patella occurred, without involvement of the enthesophyte.

**Table 2:** The results of group analysis between patellar avulsion injury versus none, including two tailed *p* values

		Patellar avulsion?		
		Yes (*n* = 26)	No (*n* = 27)	*p* value
Tendon quality	Rectus femoris	3.66	3.35	0.043*
	Vastus medialis	3.63	3.25	0.036*
	Vastus lateralis	3.69	3.35	0.020*
	Vastus intermedius	2.90	3.04	0.521

*p < 0.05 was considered statistically significant. The tendon quality was encoded as follows: 1 = normal, 2 = tendinosis, 3 = partial rupture, 4 = complete rupture

### Tendon tear location

**Fig. 3:**
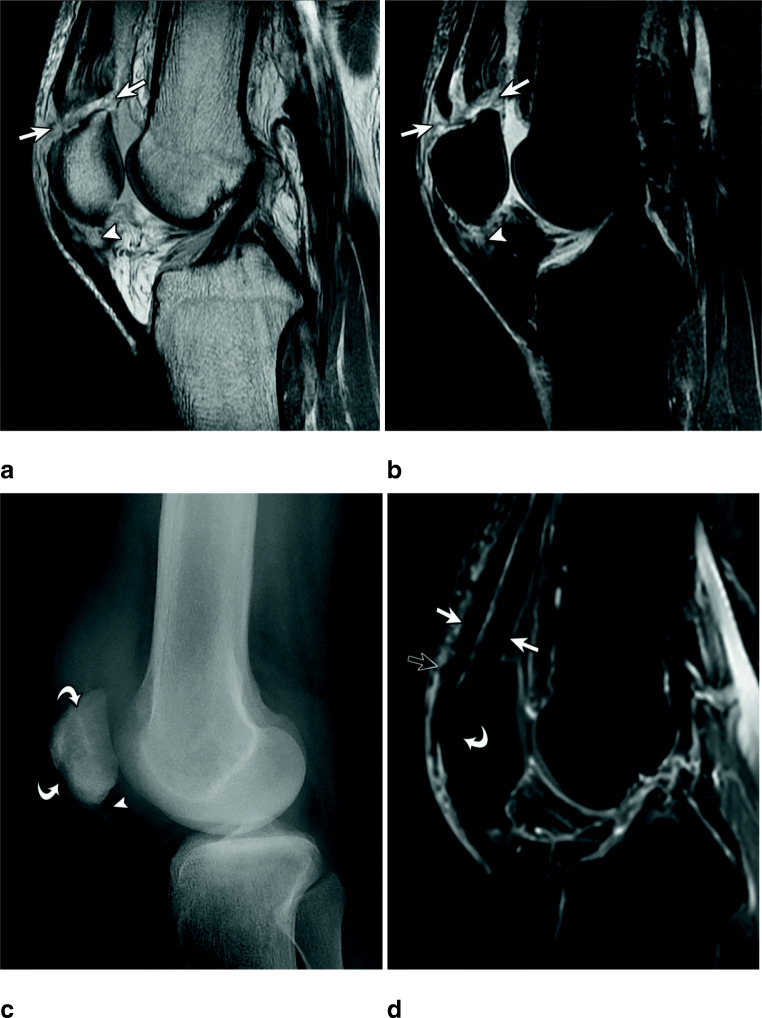
Thirty-three-year-old male with distal quadriceps femoris tear. Intermediate-weighted MR images without (**a**) and with (**b**) fat saturation show complete quadriceps tendon rupture at the osseotendinous junction (arrows). Note partial tear of the proximal patellar tendon (arrowhead). The patient was treated surgically with transpatellar bone tunnel tendon fixation (curved arrows) shown on the four weeks postoperative follow-up lateral knee radiograph (**c**). In addition, an ossification at the site of the proximal patellar tendon tear is seen (arrowhead). Sagittal T1 fat-saturated MR imaging (**d**) performed 8 years after surgery shows an intact quadriceps tendon (arrows), postsurgical susceptibility artifacts (open arrow), and a completely ossified surgical tunnel (curved arrow)

**Fig. 4:**
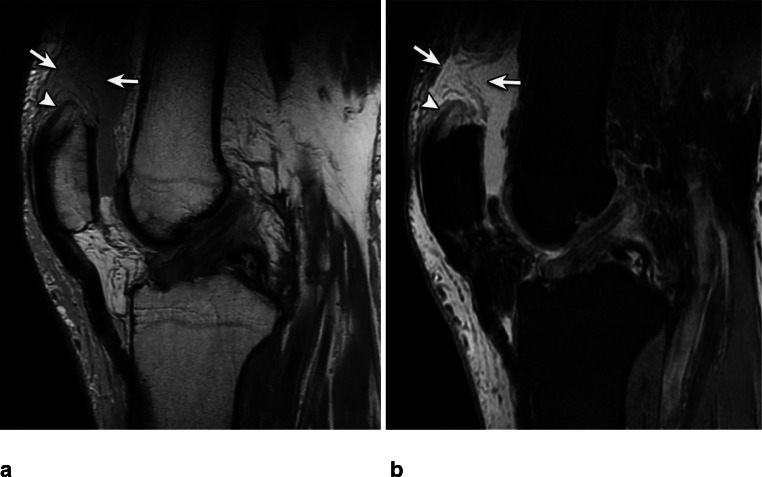
Eighty-three-year-old male with quadriceps femoris tear. Intermediate-weighted MR images without (**a**) and with (**b**) fat saturation show a complete quadriceps tendon rupture proximal to the patella (arrows) with a tendon stump (arrowhead). The patient was treated surgically with transpatellar bone tunnel tendon fixation

**Fig. 5:**
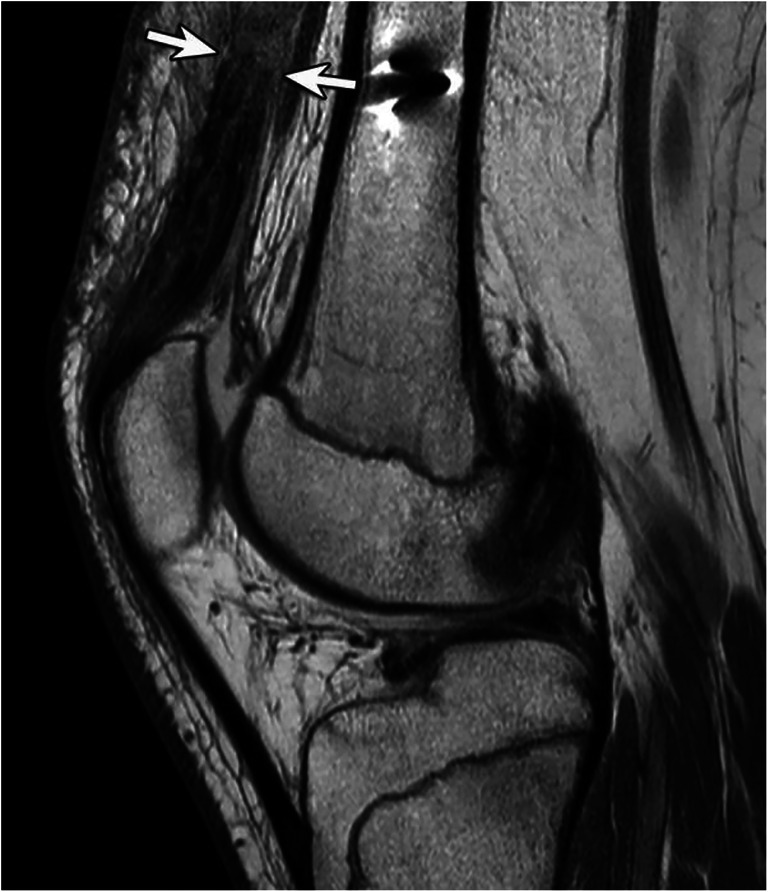
Sixteen-year-old male with quadriceps femoris tear. Intermediate-weighted MR image without fat saturation shows a complete quadriceps tendon rupture at the myotendinous junction (arrows). The patient was treated surgically with an end-to-end tendon repair

**Fig. 6:**
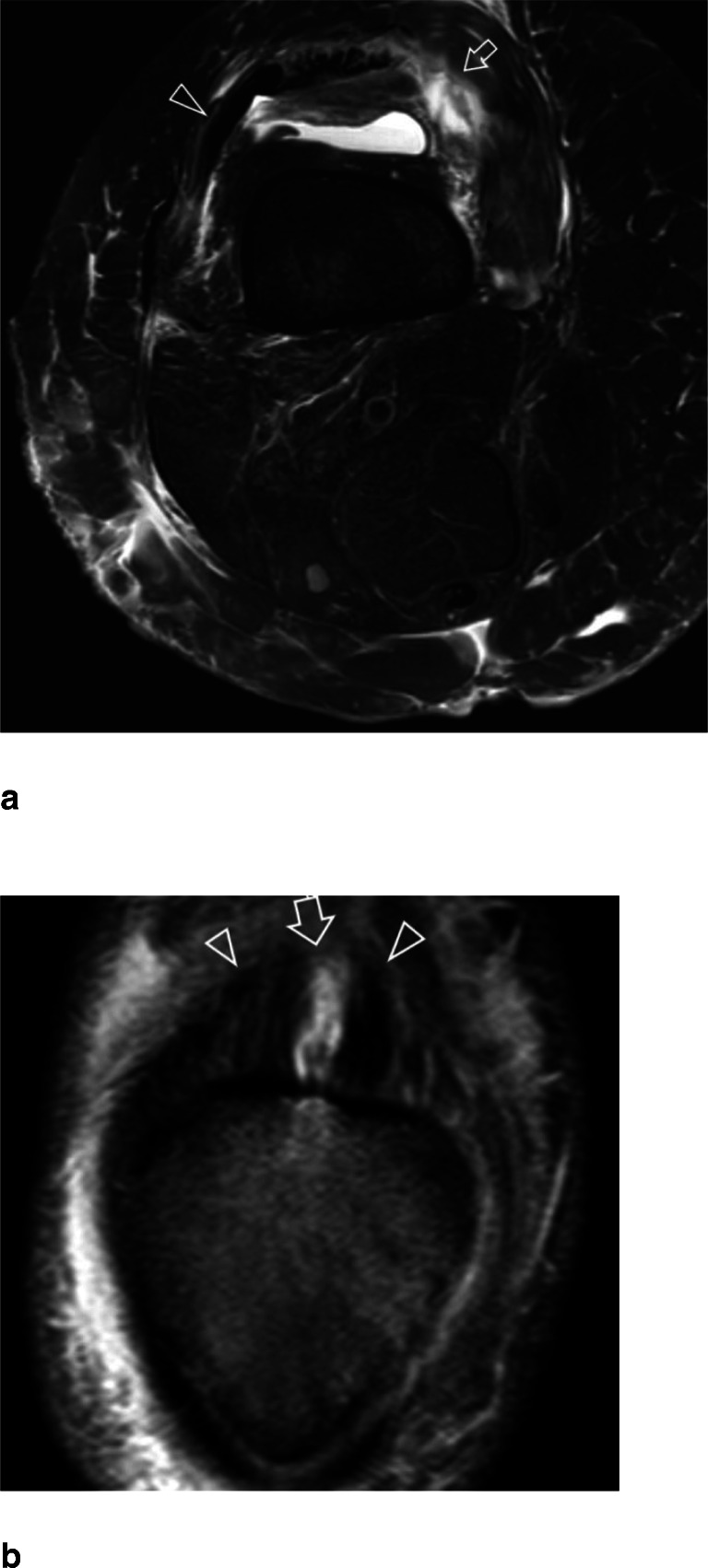
Fifty-two-year-old female with partial rupture of the right quadriceps femoris tendon (**a**). Axial T2-weighted MR image with fat saturation shows a complete disruption of the vastus medialis tendon (arrow), while the vastus lateralis tendon is intact (arrowhead). **b** Forty-seven-year-old male with partial rupture of the quadriceps femoris tendon. Coronal Intermediate-weighted MR image with fat saturation shows a partial tear of the rectus femoris tendon (arrow), while the lateral and medial portion of the tendon is intact (arrowheads)

On MR imaging, we found in total 183 tendon ruptures of all subtendons (RF, VM, VL, and VI) of the quadriceps femoris tendon. Out of these, reader 1 found 180 and reader two 171 subtendon ruptures. The VL tendon tore (including partial and complete tears) most frequently with 49 out of total 183 subtendon ruptures (26.8%). The RF incurred 47/183 ruptures (25.7%), the VM 46/183 (25.1%), and the VI tore least commonly with 41/183 tendon ruptures (22.4%). Twelve cases showed a complete tear of all tendon layers, while 13 cases showed a complete tear of the RF, VM, and VL, but without the VI. Most tendons tore either at the bone insertion (Fig. 3) or proximal to the patella (Fig. 4) (RF 44.7% and 48.9% respectively; VM 47.8% and 37.0%; VL 40.8% and 44.9%; VI 36.6% and 56.1%), creating a total of 84.8 to 93.6% cases rupturing in either of these locations. Just one case showed a rupture close to the myotendinous junction (Fig. 5). Distribution of individual tendon rupture locations is listed in Table 3 and additional examples are depicted in Figs. 2 and 6. The inter-reader agreement for rupture location was substantial (κ = 0.624–0.688).

**Table 3:** The location of the tear for each tendon is shown as a percentage of the total. Cohen’s Kappa inter-reader agreement is listed in the last row

	Rectus femoris	Vastus medialis	Vastus lateralis	Vastus intermedius
Bony avulsion	4.3% (2/47)	13.0% (6/46)	12.2% (6/49)	4.9% (2/41)
Directly at the patella	44.7% (21/47)	47.8% (22/46)	40.8% (20/49)	36.6% (15/41)
Proximal to the patella	48.9% (23/47)	37.0% (17/46)	44.9% (22/49)	56.1% (23/41)
Myotendinous junction	2.1% (1/47)	2.2% (1/46)	2.1% (1/49)	2.4% (1/41)
Inter-reader agreement	κ = 0.688	κ = 0.624	κ = 0.678	κ = 0.640

If a bony avulsion was present on radiographs, the VM and VL tendons most often tore at the bone insertion (*p* = < 0.001–0.011), while the other tendons showed no predilection for tear site (Fig. 2).

If a tendon stump was present, the tendon tear gap ranged 0.4–3.1 cm (average 1.4 cm) for the RF (*p* = 0.003), 0.4–4.1 cm (average 1.3 cm) for the VM (*p* = 0.031), 0.3–4.8 cm (average 1.2 cm) for the VL (*p* = 0.233), and 0.3–3.4 cm (average 1.5 cm) for the VI tendon (*p* = 0.028).

If retraction of the torn quadriceps tendon layers was present, retraction length ranged from 0.5 to 5.9 cm (average 2.7 cm) for the RF (*p* = 0.068), 0.4 to 6.0 cm (average 2.5 cm) for the VM (*p* = 0.023), 0.4 to 6.0 cm (average 2.5 cm) for the VL (*p* = 0.095), and 0.9 to 5.4 cm (average 2.3 cm) for the VI tendon (*p* = 0.600).

### Additional findings

The prepatellar quadriceps tendon continuation was abnormal (e.g., detached from the patella) in 43% of cases (23/53), with a substantial agreement of κ = 0.729.

### Patient records and MR imaging correlation

Twenty-seven (27/53) cases (51%) were treated surgically. Twenty-six (96%) out of 27 surgically treated cases received a transpatellar bone tunnel tendon fixation (Fig. 3c, d), and one case (1/27 or 4%) received direct end-to-end tendon repair. The surgically treated patients had complete tears of at least 2 of the quadriceps tendon layers: 5 cases showed ruptures of two tendons, 10 cases of three tendons, and 12 cases had a rupture of all four components of the quadriceps tendon.

Twenty-six (26/53) cases (49%) were treated conservatively, e.g., T-scope brace, physical therapy, and pain medication. Fourteen (14/26) patients showed only partial tendon tears of one or multiple quadriceps tendon layers and were treated conservatively. The twelve remaining conservatively managed cases consisted of two cases with one subtendon rupture, four cases with two subtendon ruptures, five cases with three subtendon ruptures, and one case with complete quadriceps tendon rupture including all 4 subtendons. In these cases, the decision for conservative treatment was based on a number of factors: the remaining clinical function of the patient’s knee, the patient’s co-morbidities which may not have allowed surgery in the risk:benefit calculation, and/or the patient’s decision against surgical treatment.

No patient had diabetes type 1, and 25% (13/52) of patients had diabetes type 2. Seven patients (7/52; 13%) had gout. One patient had a collagen disorder, i.e., Sjogren’s syndrome. Four patients (4/52; 8%) had a vascular disorder, i.e., peripheral vascular disease, venous insufficiency, Raynaud’s disease, and rheumatoid aortitis. Forty-nine (49/53) cases (92%) had a low-energy trauma, and four (4/53) cases (8%) a high-energy trauma.

## Discussion

Rupture of the quadriceps tendon is, although a well-recognized entity, not a common pathology with an incidence of just 1.37/100,000 per year [21]. The diagnosis of a quadriceps tendon rupture is usually made clinically. Radiographs are performed to assess bony avulsions, and ultrasound and MR imaging are performed if the clinical diagnosis is unclear [8].

The most important findings of the present case series study are that the superficial (25.7%) and middle layers (25.1–26.8%) of the quadriceps tendon ruptured more frequently than the deep layer (22.4%). The tendon layers ruptured more frequently in proximity to the patella (84.8–93.6% of cases). A bony avulsion was associated with higher-grade tears of the RF, VM, and VL (*p* = 0.020–0.043), but not the VI.

McMaster et al [22] showed in adult rabbits that approximately 50% of quadriceps tendon’s fibers had to be severed for complete rupture to occur; however, rupture was possible under lesser loads at areas of weakness. These weaker areas may have different abilities to handle load, such as the osseotendinous junction [22] or at hypovascular zones [4], suggesting that vascular disturbances may play a role in tendon degeneration and rupture. Yepes et al [4] described in human cadavers a hypovascular zone 1–2 cm above the patella which might be a weak point prone to rupture. Among others, they postulated that this is the most frequent point of ruptures [1, 4, 16]; however, other authors called ruptures at the osseotendinous junction to be most common [6, 13–15]. Our study shows that rupture distribution in between these two locations varies for the different tendons (84.8 to 93.6% of cases in either of these locations). Just one case in our study had a rupture close to the myotendinous junction.

Accurate clinical characterization of a quadriceps femoris tear is critical for surgical planning, in cases where surgery is indicated. With midsubstance tears where there are adequate proximal and distal tendon stumps remaining, a primary end-to-end repair can be completed using a Krackow or equivalent technique at both the proximal and distal ends of the tendon [6, 23]. Tendon tears that occur at the osseotendinous junction or with an insufficient stump are not amenable to direct end-to-end repair, favoring transpatellar bone tunnels for tendon fixation [6, 23].

A total of 96% (26 of 27) of surgically treated patients in our study received a transpatellar bone tunnel tendon fixation, while only one patient (4%) received a direct end-to-end tendon repair. This correlates with the numbers of proximal and myotendinous junction ruptures in our study; therefore, the most important information for surgical planning is not whether the rupture occurs directly at the bone versus with a residual tendon stump, but instead information pertaining to the quality of the tendon stump. Our study results indicate that ruptures in proximity to the patella do not tend to yield sufficient stump size and quality to allow for end-to-end repair and would favor transpatellar bone tunnel tendon fixation.

A bony avulsion was associated with higher-grade tears of the RF, VM, and VL (*p* = 0.020–0.043), but not the VI; therefore, scrutiny should be given when interpreting radiographs, since even with typical signs and symptoms, up to 50% of cases have reportedly been misinterpreted in the emergency department [24]. Radiographs can depict not only suprapatellar enthesophytes but also patellar bone avulsions [16], which can indicate tendon rupture. In a complete rupture, radiographs may provide additional signs such as abnormal patellar position owing to an unopposed pull from the patellar tendon distally. Follow-up MR imaging may distinguish between complete or partial quadriceps tendon tears as well as give further information regarding the intra-articular state of the knee, although small bone avulsions may be difficult to identify on MR images [16].

In our investigation, the superficial (25.7%) and middle layers (25.1–26.8%) of the quadriceps tendon ruptured more frequently than the deep layer (22.4%). This corroborates findings from Scuderi et al [1], but is counter to another study by Raatikainen et al [17]; however, the first study examined only 20, and the latter 28 tendon ruptures compared to 53 in our study.

Our surgically treated patients all had a minimum of two subtendons of the quadriceps femoris torn. This stresses the importance of searching for the precise extent of affected tendons in cases of partial rupture, as those patients with one subtendon tear are often managed conservatively. However, since not all patients with a clinically suspected and surgically treated quadriceps tendon rupture receive preoperative MR imaging, we cannot yet conclude that two complete tendon ruptures are a certain cut-off for surgical treatment, especially since clinical parameters often play an important role in treatment planning, e.g., rest-function of the quadriceps tendon and co-morbidities.

We acknowledge limitations in our study. This was a retrospective study; however, a quadriceps tendon tear is so rare that a prospective study would hardly be feasible without a protracted study timeline; patients in our study were imaged over an 18-year period. Due to the retrospective nature of the study, only patients with MR imaging were included. However, a greater number of patients might have received treatment without imaging or other imaging modalities. The electronic medical records over the past 18 years were not sufficient to evaluate further clinical risk factors and co-morbidities for quadriceps tendon ruptures. Though we were able to retrospectively elaborate the type of therapy used in our study population, surgical reports were not uniformly detailed enough to elucidate precise rupture location, i.e. measurements, and quadriceps tendon layer involvement.

## Conclusion

The trilaminar quadriceps femoris tendon tends to tear at its superficial and middle layers more frequently than its deep layer, and usually in proximity to the patella. When present, an associated patellar bony avulsion portends higher severity of a quadriceps tendon tear. Our study results also indicate that ruptures in proximity to the patella do not tend to yield sufficient stump quality to allow for end-to-end repair and would favor transpatellar bone tunnel tendon fixation. Precise tear characterization by high-resolution MR imaging allows for more accurate diagnosis and a tailored approach to treatment selection.

## Abbreviations

MR: Magnetic resonance
RF: Rectus femoris
VI: Vastus intermedius
VL: Vastus lateralis
VM: Vastus medialis

## Declarations

Institutional Review Board approval was obtained by the Medical School Institutional Review Board (IRBMED) of the University of Michigan for this retrospective study, and informed consent was waived.

### Guarantor

Jon A. Jacobson, MD, Department of Radiology, University of Michigan, 1500 East Medical Center Drive, Ann Arbor, Michigan, 48103, USA, jjacobsn@med.umich.edu

### Statistics and biometry

No complex statistical methods were necessary for this paper.

### Methodology

• retrospective

• observational

• performed at one institution
